# *N,N*′-Bis(2-cyclohexylethyl)naphtho[2,3-*b*:6,7-*b*′]dithiophene Diimides: Effects of Substituents

**DOI:** 10.3390/molecules21080981

**Published:** 2016-07-28

**Authors:** Masahiro Nakano, Daisuke Hashizume, Kazuo Takimiya

**Affiliations:** 1 Emergent Molecular Function Research Group, RIKEN Center for Emergent Matter Science (CEMS), 2-1 Hirosawa, Wako, Saitama 351-0198, Japan; takimiya@riken.jp; 2 Materials Characterization Support Unit, RIKEN Center for Emergent Matter Science (CEMS), 2-1 Hirosawa, Wako, Saitama 351-0198, Japan; hashi@riken.jp

**Keywords:** application of naphthalene diimide, organic synthesis, supramolecular chemistry, organic field-effect transistor, crystal structure

## Abstract

Naphtho[2,3-*b*:6,7-*b*′]dithiophene-4,5,9,10-tetracarboxylic diimide (NDTI) is a promising electron-deficient building block for n-type organic conductors, and the performance of NDTI-based field-effect transistors (FETs) is largely dependent on the substituents that alter the supramolecular organization in the solid state and, in turn, the intermolecular orbital overlap. For this reason, the rational selection of substituent on imide nitrogen atoms and/or thiophene α-positions is the key to developing superior n-type organic semiconductors. We here report new NDTI derivatives having *N*-(2-cyclohexylethyl) groups. Despite their one-dimensional packing structures in the solid state regardless of the presence or absence of chlorine groups at the thiophene α-positions, their FETs show promising performance with electron mobilities higher than 0.1 cm^2^·V^−1^·s^−1^ under ambient conditions. We also discuss how the cyclohexylethyl groups affect the packing structure in comparison with analogous *n*-octyl derivatives having the same number of carbon atoms.

## 1. Introduction

**Figure 1:**
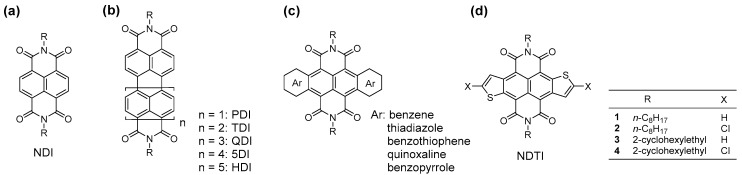
Molecular structures of semiconducting molecules based on naphthalene diimide (NDI, **a**); rylene diimides (**b**); core-extended NDIs (**c**); and naphtho[2,3-*b*:6,7-*b*′]dithiophene-4,5,9,10-tetracarboxylic diimide (NDTI, **d**).

Rylene diimides, represented by naphthalene diimide (NDI, Figure 1a), have been the focus of studies in the past few decades because of their potential use in chromophores, supramolecules, optoelectronic materials, and n-type organic semiconductors [1,2,3,4]. On the one hand, the optical properties of the rylene diimide-based system can be tuned by the central rylene moiety [5,6], i.e., increasing the number of naphthalene moieties (Figure 1b) can drastically alter the absorption range from ultraviolet to visible and to the infrared region. On the other hand, the lateral extension of the π-conjugation system based on NDI, the smallest rylene diimide molecule, has also been examined by fusing different aromatic rings, such as benzene [7,8,9], thiophene [10], thiazole [11], benzo[*b*]thiophene [12], quinoxaline [13,14], benzo[*b*]pyrrole [15], and so on (Figure 1c). In contrast to the former vertical π-extension, the latter approach is a promising way of controlling the electronic structure of the resulting core-extended NDI derivatives because the lowest unoccupied molecular orbital (LUMO) of the system tends to localize on the NDI skeleton, i.e., the vertical molecular axis, whereas the highest occupied molecular orbital (HOMO) tends to delocalize in the lateral direction through the naphthalene 2-, 3-, 6-, and 7-carbon atoms. This affords an opportunity to finely tune the HOMO while keeping the LUMO almost intact or only slightly altering it [15].

Naphtho[2,3-*b*:6,7-*b*′]dithiophene-4,5,9,10-tetracarboxylic diimide (NDTI, Figure 1d) is an example of core-extended NDI derivatives. We have been interested in the NDTI structure for the following reasons [16,17]: (i) It has “chemical flexibility”, which enables chemical modifications of both the imide nitrogen atoms and the thiophene α-positions. The modification of the imide nitrogens can be used to enhance solubility and/or supramolecular organization in the solid state of the resulting materials, and the substitution at the α-positions of fused thiophenes can be used to tune the electronic structure of the resulting systems by both inductive and resonance effects [18,19,20,21]; (ii) It possesses a planar structure, which is beneficial for carrier transport owing to the less sterically demanding fused-thiophene rings without β-substituents; (iii) It has slightly lower LUMO energy levels (*E*_LUMO_s: ~ −4.0 eV) and smaller HOMO-LUMO gaps than the corresponding NDI derivatives. In fact, with the NDTI core structure, a range of optoelectronic materials, e.g., n-type organic semiconductors, and ambipolar polymers and oligomers for field-effect transistors (FETs) and solar cell applications, have been developed [16,17,18,19,20,21].

The key to developing superior organic semiconductors for n-type FET applications is to realize low-lying *E*_LUMOS_ (−4.0 eV or below) and a molecular packing structure with strong intermolecular interaction. The NDTI derivatives generally meet the former criterion as they have inherently low-lying *E*_LUMO_s, as mentioned above. For the latter criterion, the chemical flexibility of NDTI derivatives would enable us to tune the supramolecular organization in the solid state. Herein, we report the synthesis and characterization of NDTI derivatives whose imide nitrogen atoms are modified with relatively bulky 2-cyclohexylethyl groups, namely, *N,N*′-bis(2-cyclohexylethyl)NDTIs (Figure 1, **3** and **4**), as small-molecule n-type organic semiconductors.

## 2. Results

### 2.1. Molecular Design and Synthesis

Among the many NDI derivatives examined as the active material in thin-film transistors (TFTs), *N*,*N*′-dicyclohexyl NDI (CH-NDI) is one of the most promising because it affords high electron mobility (up to 6 cm^2^·V^−1^·s^−1^) in TFT devices [22,23]. CH-NDI has a two-dimensional (2D) π-stacking structure, often referred to as a “brick layer” structure that is similar to the molecular arrangements of 6,13-bis(triisopropylsilylethynyl)pentacene (TIPS-pentacene) and related compounds [24]. In fact, theoretical calculations of intermolecular LUMO overlap based on the crystal structure have demonstrated that the molecular arrangement is 2D with relatively large intermolecular LUMO overlaps [25], corroborating the reported high electron mobility in the TFT devices. On the other hand, *N,N*′-dioctyl-NDTI (C_8_-NDTI, **1**, Figure 1), the first NDTI derivative examined as an n-type organic semiconductor, shows electron mobility of up to 0.05 cm^2^·V^−1^·s^−1^. The relatively low mobility of **1**-based devices can be explained by its one-dimensional (1D) π-stacking columnar structure, which is not necessarily suitable for high-performance TFTs, and by the polymorphism in the thin-film state. The out-of-plane X-ray diffraction (XRD) patterns of vapor-deposited **1** thin films showed two series of peaks, one of which could be assigned to the bulk single-crystal phase and the other, to an unknown phase [16].

**Figure 2:**
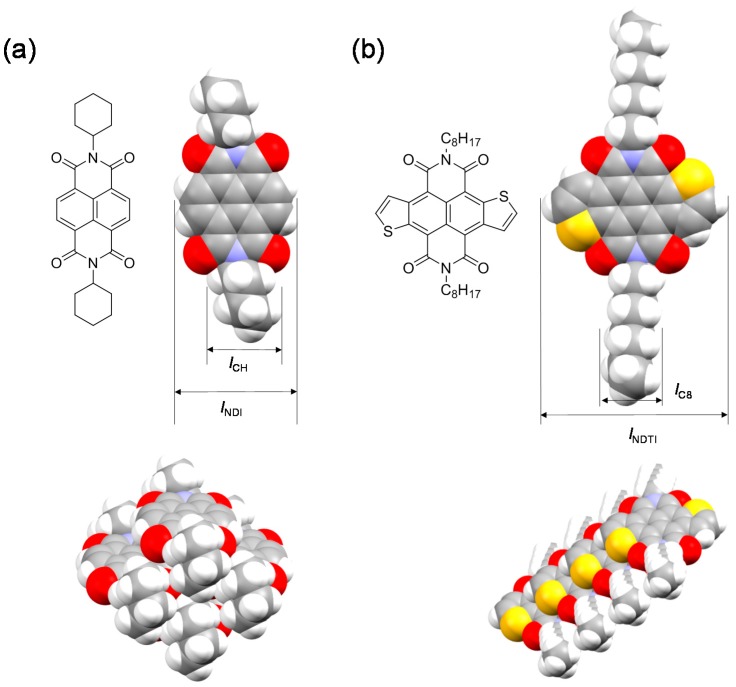
Size comparison of the π-core and the alkyl group and packing structures of *N*,*N*′-dicyclohexyl NDI (CH-NDI) (**a**) and **1** (**b**) extracted from their single-crystal X-ray structures: *l*_NDI_ = ca. 6.4 Å, *l*_CH_ = 3.0 (4.2) Å, *l*_NDTI_ = ca. 11.2 Å, *l*_C8_ = 2.0 (2.5) Å.

**Scheme 1:**
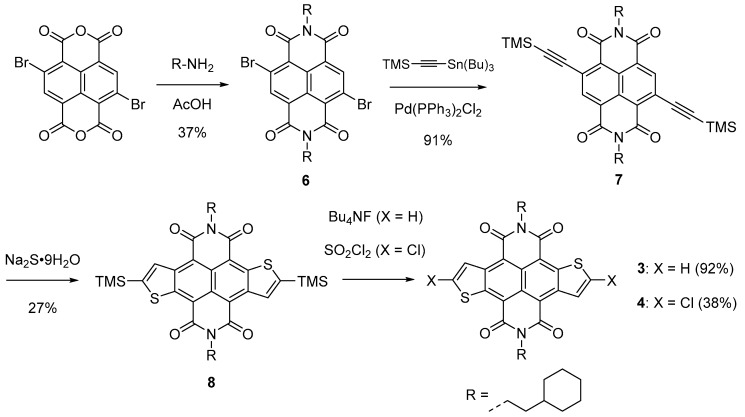
Synthesis of *N,N*′-bis(2-cyclohexylethyl)-NDTI derivatives (**3** and **4**).

With these results of **1**, we posed a hypothesis for a molecular design strategy using the NDTI core structure in order to develop improved n-type organic semiconductors while taking advantage of the low-lying LUMO of NDTI. As demonstrated by Anthony, the relative sizes of the π-core and the substituents could play a critical role in determining the packing structure in the solid state [26]. In the present NDI/NDTI cases, the ratio of the size of the π-core, i.e., naphthalene (NDI) or naphtho[2,3-*b*:6,7-*b*′]dithiophene (NDTI), to the size of the *N*-alkyl group, i.e., cyclohexyl (CH) or *n*-octyl (C_8_), should be taken into account (Figure 2). In contrast to the CH-NDI combination, the C_8_ groups seemed to be too narrow compared to the wide NDTI core in **1**. This imbalanced combination of the alkyl group and the π-core in the C_8_-NDTI combination would make the molecule less favorable for 2D π-stacking and rather favorable for 1D π-stacking. In addition, the large difference in volume between the π-core and the alkyl group in C_8_-NDTI would afford several thermodynamically stable packing structures that would cause undesirable polymorphism in the vapor-deposited thin film. Therefore, we anticipated that relatively large cyclohexyl substituents would be promising for the NDTI core to induce the 2D π-stacking structure in the solid state, and attempted to synthesize *N,N*′-dicyclohexyl-NDTI. However, the introduction of the cyclohexyl groups to 2,6-dibromonaphthalene carboxylic anhydride (Scheme 1), a key starting material for NDTI derivatives, met with failure; undesirable nucleophilic substitution reaction took place at the 2- and/or 6-positions of the naphthalene core, probably owing to the high nucleophilicity of cyclohexyl amine [27]. Thus, instead of cyclohexyl groups, we turned our attention to 2-cyclohexylethyl groups to examine the substituent effect on the packing structure.

The synthesis of 2-cyclohexylethyl-substituted NDTI derivatives is demonstrated in Scheme 1. The synthesis is basically the same as that of C_8_-NDTIs (**1** and **2**). The reaction of readily available 2,6-dibromonaphthalene tetracarboxylic anhydride and 2-cyclohexylethylamine gave corresponding diimide (**6**) in 37% isolated yield. After the introduction of trimethylsilyl (TMS)-acetylene moiety via a Stille coupling reaction to give **7**, the sodium-sulfide-induced thiophene annulation reaction afforded desired NDTI derivative (**8**) with TMS groups at the thiophene α-positions in 27% yield. The TMS groups could be readily desilylated with tetrabutylammonium fluoride or chlorinated with sulfuryl chloride to afford α-unsubstituted (**3**) or chlorinated NDTI derivative (**4**), respectively, in reasonable yields. Chlorinated NDTI derivative (**4**) was synthesized because the corresponding C_8_-NDTI derivative with two chlorine substituents (**2**) showed the highest field-effect electron mobility (ca. 0.8 cm^2^·V^−1^·s^−1^) among the NDTI-based organic semiconductors, and it was unveiled that the chlorine groups induced additional intermolecular interaction to realize a 2D π-staking structure in the solid state [16]. These new NDTI derivatives were fully characterized by spectroscopic analysis as well as single-crystal X-ray analysis (vide infra).

### 2.2. Physicochemical Properties

**Figure 3:**
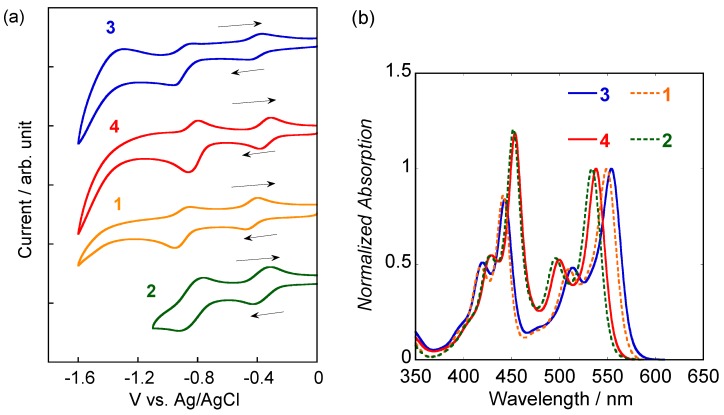
Cyclic voltammograms (**a**) and UV-vis absorption spectra (**b**).

The electronic structures of newly synthesized 2-cyclohexylethyl-NDTI derivatives (**3** and **4**) were examined by cyclic voltammetry and UV-vis spectroscopy (Figure 3). Both electrochemical and optical properties of the 2-cyclohexylethyl-NDTI derivatives are quite similar to those of the C_8_-NDTI derivatives (**1** and **2**) (Table 1), indicating that the electronic effect of the 2-cyclohexylethyl group is almost the same as that of the *n*-octyl group. The chlorine groups at the thiophene α-positions induced anodic shifts of reduction waves owing to their electron-withdrawing nature. As a result, their *E*_LUMO_s estimated from the reduction onset are 4.0 and 4.1 eV below the vacuum level, respectively, which meet the criteria for stable electron transport in n-type organic FETs [28].

### 2.3. Single-Crystal Structures

**Figure 4:**
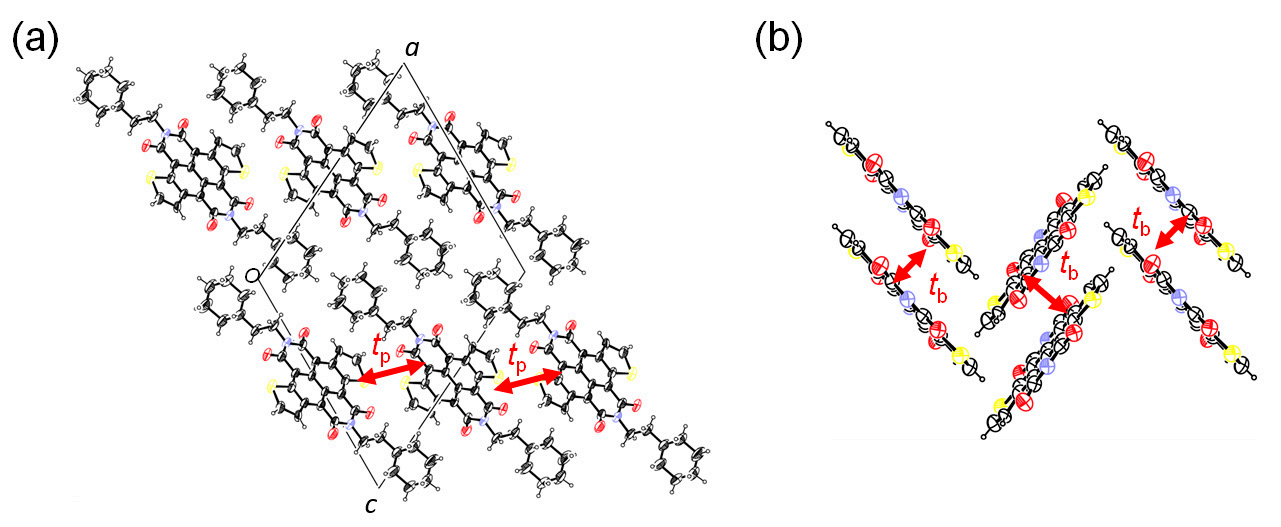
Crystal structure of **3**: (**a**) *b*-axis projection and (**b**) side view of stacking columnar structure. Calculated transfer integrals of lowest unoccupied molecular orbitals (LUMOs), *t*_p_ and *t*_b_, are 17 and 90 meV, respectively.

One of the reasons for introducing the 2-cyclohexyl group as the relatively bulky alkyl group rather than the *n*-octyl group is that we would like to know its effect on the packing structure. Figure 4 shows the packing structure of **3** elucidated by single-crystal X-ray analysis. Different from our initial expectations, the packing structure of **3** is classified into the π-stacking columnar structure (π-π distance: 3.47 Å), and is very similar to that of **1**. Although the space group and the direction of the unit cell of **3** are different from those of **1**, the mutual positions of the π-stacking columns are basically the same (Figure 4a); the molecular π-plane in the neighboring columns in the [1 0 1] direction tilts in the opposite direction, resulting in the perpendicular molecular arrangement in the adjacent columns (Figure 4b). This may indicate that even with the 2-cyclohexylethyl group, which is bulkier than the *n*-octyl group, the balance between the sizes of the NDTI core and the substituents is not suitable for realizing the 2D π-stacking (Figure 2a). The calculated transfer integrals of LUMOs (orbital overlaps) between the molecules in the stacking columns (*t*_b_) are 90 meV, whereas those between the columns (*t*_p_) are 17 meV [29]. These values are comparable to those calculated for **1** (75 and 16 meV, respectively), indicating that **3** has a similar 1D electronic structure in the solid state to **1**.

**Figure 5:**
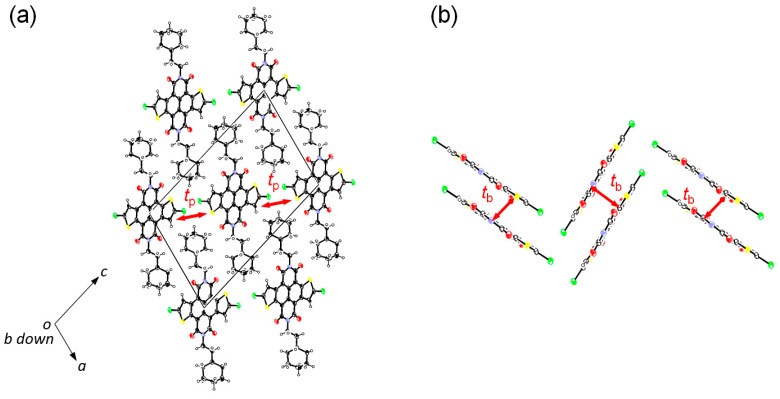
Crystal structure of **4**: (**a**) *b*-axis projection and (**b**) side view of stacking columnar structure. Calculated transfer integrals of LUMOs, *t*_p_ and *t*_b_, are 8 and 105 meV, respectively.

Depicted in Figure 5 is the crystal structure of **4**, which is, at a glance, similar to that of **3**. In fact, the molecules of **4** form a stacking columnar structure along the *b*-axis direction with the π-π distance of 3.40 Å (Figure 5a), and the molecules in the adjacent columns tilt in the opposite direction (Figure 5b). This packing motif is markedly different from that of **2**, which has a characteristic 2D π-stacking structure [17]. In the crystal structure of **2**, the chlorine atoms likely play an important role in defining the crystal structure. The intermolecular Cl-O=C interactions form ribbon-like molecular arrays through the neighboring columns, where all the π-planes of the NDTI cores are on the same plane, and the slight molecular offset in the stacking column creates a weak but substantial π-π overlap in the diagonal direction between the molecules in the neighboring columns, resulting in a quasi 2D electronic structure. In the crystal structure of **4**, the chlorine atoms also participate in the intermolecular interactions; however, the chlorine atoms in **4** directly interact with the chlorine atoms of the other molecules in the adjacent columns. The intermolecular Cl-Cl distance is 3.49 Å, which is slightly shorter than the sum of van der Waals radii (3.50 Å) [30]. As is often observed for “chlorine bonds” in organic crystals [31], the chlorine atoms are oriented perpendicular to each other, and this serves as one of the driving forces that enable **4** to form the 1D π-stacking structure.

The calculated transfer integrals between the molecules in the stacking columns (*t*_b_) and between the columns (*t*_p_) are 105 and 8 meV, respectively, indicating that the electronic structure of **4** is largely one-dimensional. The anisotropy of the orbital overlaps is much larger than those of **1** and **3**; the ratio of the transfer integrals for the intra- and inter-stacking directions, *t*_b_/*t*_p_, is ca. 4.7, 5.3, and 13 for **1**, **3**, and **4**, respectively. The higher anisotropy of **4** than those of **1** and **3** can be explained by the shorter π-π stacking distance of **4**, which enhances the orbital overlap in the stacking direction, and the existence of inter-columnar Cl-Cl interactions, which may reduce the LUMO-LUMO overlap owing to the fact that the chlorine atoms marginally contribute to the LUMO of **4**. From the results of crystal structure analyses, the cyclohexyl group is not suitable for realizing the 2D π-stacking structure that was observed for CH-NDI [22].

### 2.4. Thin-Film Deposition and Field-Effect Transistors

**Figure 6:**
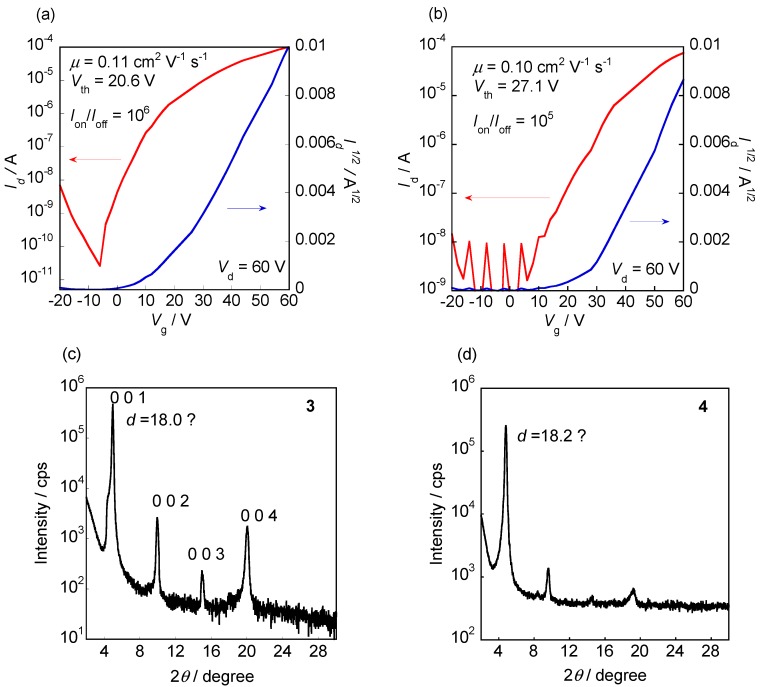
Transfer characteristics of **3**- (**a**) and **4**- (**b**) based n-type thin-film transistors (TFTs) evaluated under ambient conditions, and out-of-plane X-ray diffraction (XRD) patterns of vapor-deposited thin films of **3** (**c**) and **4** (**d**) on the Si/SiO_2_ substrate at 150 *°*C.

Thin films of **3** and **4** were deposited by vapor deposition on Si/SiO_2_ substrates modified with octadecyltrichlorosilane (ODTS), and were kept at 150 or 200 °C in order to obtain crystalline thin films. TFTs with the bottom-gate, top-contact (BGTC) configuration were then fabricated by evaporating the source and drain electrodes on top of the thin films. The devices showed typical n-type FET responses under both ambient and vacuum conditions, and no significant differences in performances were observed, indicative of the stable electron transport as expected from their low-lying LUMO energy levels (Table 1) [28]. The mobilities extracted from the saturation regime (Figure 6) were as high as 0.10 cm^2^·V^−1^·s^−1^ for the devices based on both **3** and **4**, and were higher than that of **1**-based ones (~0.05 cm^2^·V^−1^·s^−1^) but lower than that of **2**-based ones (~0.8 cm^2^·V^−1^·s^−1^). The low mobilities of **3**- and **4**-based devices compared to **2**-based ones could be understood in terms of the packing structure and hence the electronic structure in the solid state. As discussed above, the electronic structures of **3** and **4** in the solid state were characterized as 1D systems, which are not suitable for realizing the high mobility in the TFT setting owing to the arbitrary orientation of crystalline grains on the substrate surface. On the other hand, the higher mobility of **3**- and **4**-based devices than **1**-based ones, despite the fact that all the electronic structures of **1**, **3**, and **4** in the solid state are 1D systems, is rather intriguing. This could be explained by their distinct crystallinity in the thin-film state; as clearly observed in the thin-film XRD patterns, **3** thin film showed a series of peaks assignable to (0 0 l) reflection with the d-spacing of ca. 18 Å for **3** based on the single-crystal X-ray analysis, whereas for **4** thin film, no clear assignment could be made for the series of peaks with the d-spacing of 18.2 Å (Figure 6c,d) in the single-crystal structure. The appearance of only one series of peaks is in sharp contrast to **1** thin film in which regardless of the substrate temperature during deposition, two series of peaks appear, one of which can be assigned to 0 0 l from the single-crystal X-ray data.

## 3. Discussion

As demonstrated by the single-crystal X-ray analyses, the packing structures of **3** and **4** are classified into 1D π-stacking, similar to that of **1**. This is very different from what we had initially expected from the substitution of the 2-cyclohexylethyl group for the *n*-octyl group. The results, on one hand, may imply that the crystal engineering approach that involves modifying substituents on the NDTI core is not straightforward. On the other hand, even with the 1D π-stacking structure, TFT devices with **3** and **4** thin films as the active layer showed higher electron mobility than **1**-based devices. The thin-film XRD offers a clear explanation of the improved mobility: no polymorphism was observed for the **3** and **4** thin films, whereas the **1** thin film consisted of at least two different crystal phases. It is interesting to point out that **1** and **3** have almost the same molecular formula and molecular weight (**1**: C_34_H_38_N_2_O_4_S_2_; Mw: 602.78, **3**: C_34_H_34_N_2_O_4_S_2_; Mw: 598.77). Although they are not crystallographically identical, their structures are vertically the same in terms of molecular arrangement and cell dimensions. By comparing the structural parameters in detail, we notice that the cell volume of **3** (*V* = 1419.2 Å^3^) is smaller than that of **1** (*V* = 1485 Å^3^) by ca. 4.5%, although the molecular weight difference is less than 1%; as a result, the density of **3** (*d* = 1.406 g·cm^−3^) is higher than that of **1** (*d* = 1.348 g·cm^−3^) by ca. 4.2%. This clearly means that **3** is a more densely packed crystal than **1**, indicating that the substitution of the 2-cyclohexylethyl group for the *n*-octyl group can enhance the packing efficiency in the solid state. In other words, the flexible *n*-octyl group in **1** forms a loose crystal packing that can induce several thermodynamically stable packing structures on the substrate. Thus, we conclude that although the 2D π-stacking structure is not realized with the 2-cyclohexylethyl groups on the NDTI core, its rigid and less flexible nature can suppress polymorphism in the thin-film state, leading to the enhanced mobility in TFT devices. That such a subtle change in the alkyl group can alter the device characteristics is indeed an interesting aspect of organic semiconductors. We have not yet elucidated the effect of the substituent at the thiophene α-position of the NDTI core in detail. The elaboration of novel superior n-type organic semiconductors based on the NDTI core by modifying both the imide and α-substituents is in progress in our laboratory.

## 4. Materials and Methods

### 4.1. Synthesis

All chemicals and solvents are of reagent grade. Dichloromethane was purified with a standard procedure prior to use. 2,6-Dibromonaphthalene-1,4,5,8-tetracarboxylic dianhydride was prepared according to a reported procedure [32]. Melting points were uncorrected. Nuclear magnetic resonance spectra were obtained in deuterated chloroform (CDCl_3_) or 1,1,2,2-tetrachloroethane-*d*_2_ (TCE-*d*_2_) with TMS as internal reference; chemical shifts (δ) are reported in parts per million. IR spectra were recorded using a KBr pellet. EI-MS spectra were obtained using an electron impact ionization procedure (70 eV). The molecular ion peaks of sulfur- and/or chlorine-containing compounds showed a typical isotopic pattern, and all the mass peaks are reported based on ^32^S, ^79^Br, or ^35^Cl, respectively. HRMS measurements were carried out at the Molecular Structure Characterization Unit, RIKEN Center for Sustainable Resource Science (CSRS).

*N,N′-Bis(2-cyclohexylethyl)-2,6-dibromo-1,4,5,8-naphthalene tetracarboxylic acid diimide* (**6**). A mixture of 2,6-dibromonaphthalene-1,4,5,8-tetracarboxylic dianhydride (8.32 g, 19.5 mmol) and 2-cyclohexylamine (9.93 g, 78.1 mmol) in acetic acid (500 mL) was refluxed for 3 h. The mixture was cooled and diluted with water (800 mL). The resulting slurry was filtered, and the collected solid was washed with methanol and dried in air. Recrystallization from chloroform-methanol gave *N*,*N′*-bis(2-cyclohexylethyl)-2,6-dibromo-1,4,5,8-naphthalene tetracarboxylic acid diimide (**6**) as a yellow solid (4.65 g, 37%): ^1^H-NMR (400 MHz, CDCl_3_) δ 8.96 (s, 2H), 4.22 (t, *J* = 8.0 Hz), 1.85–1.64 (m, 14H), 1.46–1.38 (m, 2H), 1.42–1.00 (m, 10H); ^13^C-NMR (100 MHz, CDCl_3_) δ 160.73, 160.68, 139.0, 128.3, 127.8, 125.5, 124.3, 39.9, 36.1, 35.3, 33.2, 26.6, 26.2; EI-MS (70 eV) = 644 (M^+^); Mp 303–305 °C; HRMS (EI) *m/z* calcd for C_30_H_32_N_2_O_4_Br_2_ [M]^+^ 642.0729. Found: 642.0718; IR (KBr) ν = 1653, 1713 cm^−1^ (C=O).

*N,N′-Bis(2-cyclohexylethyl)-2,6-bis((trimethylsilyl)ethynyl)-1,4,5,8-naphthalene tetracarboxylic acid diimide* (**7**). A mixture of *N*,*N′*-bis(2-cyclohexylethyl)-2,6-dibromo-1,4,5,8-naphthalene tetracarboxylic acid diimide (**6**) (4.50 g, 6.99 mmol), tributyl(trimethylsilylethynyl)tin (5.39 g, 13.9 mmol), and bis(triphenylphosphine)palladium(II) dichloride (700 mg, 0.7 mmol) in toluene (400 mL) was stirred under reflux condition for 3 h. After cooling, the solvent was evaporated. Recrystallization of the residue from tetrahydrofuran-methanol gave *N*,*N*′-bis(2-cyclohexylethyl)-2,6-bis((trimethylsilyl)ethynyl)-1,4,5,8-naphthalene tetracarboxylic acid diimide (7) as a yellow solid (4.31 g, 91%): ^1^H-NMR (400 MHz, CDCl_3_) δ 8.96 (s, 2H), 4.20 (t, *J* = 8.0 Hz), 1.86–1.61 (m, 14H), 1.45–1.39 (m, 2H), 1.29–1.01 (m, 10H), 0.38 (s, 18H); ^13^C-NMR (100 MHz, CDCl_3_) δ 162.0, 161.4, 138.1, 127.1, 126.5, 126.4, 125.4, 110.9, 103.6, 39.5, 36.1, 35.5, 33.4, 26.8, 26.4, −0.1; EI-MS (70 eV) = 679 (M^+^); Mp >269–270 °C; HRMS (EI) *m/z* calcd for C_40_H_50_N_2_O_4_Si_2_ [M]^+^ 678.3309. Found: 678.3307; IR (KBr) ν = 1669, 1709 cm^−1^ (C=O).

*N,N′-Bis(2-cyclohexylethyl)-2,7-bis(trimethylsilyl)naphtho[2,3-b:6,7-b′]dithiophene-4,5,9,10-tetracarboxylic acid diimide* (**8**). Under argon atmosphere, sodium sulfide hydrate (Na_2_S·9H_2_O, 8.91 g, 37.1 mmol) was added to a stirred suspension of *N*,*N*′-bis(2-cyclohexylethyl)-2,6-bis((trimethylsilyl)ethynyl)-1,4,5,8-naphthalene tetracarboxylic acid diimide (**7**) (4.2 g, 6.19 mmol) in ethanol (300 mL) and acetic acid (6 mL) at 60 °C. After stirring at the same temperature for 12 h, the mixture was stirred at room temperature for 12 h under atmospheric condition. Then, the mixture was diluted with water (ca. 300 mL). The resulting precipitate was collected by filtration and washed with methanol. After removal of methanol in vacuo, the crude product was purified by column chromatography (SiO_2_, dichloromethane/hexane, 1:2 *v*/*v*). Recrystallization from chloroform-methanol gave *N*,*N*′-bis(2-cyclohexylethyl)-2,7-bis(trimethylsilyl)naphtho[2,3-*b*:6,7-*b*′]dithiophene-4,5,9,10-tetracarboxylic acid diimide (8) as a maroon solid (1.24 g, 27%): ^1^H-NMR (400 MHz, CDCl_3_) δ 9.06 (s, 2H), 4.34 (t, *J* = 8.0 Hz, 4H), 1.92–1.68 (m, 14H), 1.56–1.47 (m, 2H), 1.36–1.04 (m, 10H), 0.54 (s, 18H); ^13^C-NMR (100 MHz, CDCl_3_) δ 163.2, 163.1, 159.5, 148.1, 144.1, 130.5, 122.4, 118.3, 117.1, 39.7, 36.4, 35.4, 33.3, 26.7, 26.4, −0.3; EI-MS (70 eV) = 742 (M^+^); Mp 309–311 °C; HRMS (EI) *m/z* calcd for C_40_H_50_N_2_O_4_S_2_Si_2_ [M]^+^ 742.2751. Found: 742.2758; IR (KBr) ν = 1649, 1686 cm^−1^ (C=O).

*N,N′-Bis(2-cyclohexylethyl)naphtho[2,3-b:6,7-b′]dithiophene-4,5,9,10-tetracarboxylic acid diimide* (**3**). *N*,*N*′-Bis(2-cyclohexylethyl)-2,7-bis(trimethylsilyl)naphtho[2,3-*b*:6,7-*b*′]dithiophene-4,5,9,10-tetracarboxylic acid diimide (**8**) (371 mg, 0.5 mmol) was dissolved in dry tetrahydrofuran (15 mL) and acetic acid (0.15 mL), and then tetra-n-butylammonium fluoride (1 M in THF, 1.5 mL) was added to the solution at 0 °C. After stirring for 2 h at room temperature, the solution was diluted with methanol (ca. 30 mL). The resulting precipitate was collected by filtration and washed with water, methanol, ethyl acetate, and hexane to give *N*,*N*′-bis(2-cyclohexylethyl)naphtho[2,3-*b*:6,7-*b*′]dithiophene-4,5,9,10-tetracarboxylic acid diimide (3) as a red solid (275 mg, 91%): ^1^H-NMR (400 MHz, TCE-*d*_2_) δ 9.11 (d, *J* = 6.0 Hz, 2H), 8.21 (d, *J* = 6.0 Hz, 2H), 4.47 (t, *J* = 8.0 Hz, 4H), 2.00–1.78 (m, 14H), 1.75–1.62 (m, 2H), 1.45–1.21 (m, 10H); ^13^C-NMR (100 MHz, TCE-*d*_2_) δ 163.3, 163.2, 145.2, 143.2, 140.1, 129.5, 124.6, 123.0, 118.4, 36.7, 36.4, 35.6, 33.4, 26.8, 26.3; EI-MS (70 eV) = 599 (M^+^); Mp 306–309 °C; HRMS (EI) *m/z* calcd for C_34_H_34_N_2_O_4_S_2_ [M]^+^ 598.1960. Found: 598.1956; IR (KBr) ν = 1645, 1689 cm^−1^ (C=O).

*N,N′-Bis(2-cyclohexylethyl)-2,7-dichloronaphtho[2,3-b:6,7-b′]dithiophene-4,5,9,10-tetracarboxylic acid diimide* (**4**). Under argon atmosphere, sulfuryl chloride (170 μL, 2.1 mmol) was added to a stirred suspension of *N*,*N*′-bis(2-cyclohexylethyl)-2,7-bis(trimethylsilyl)naphtho[2,3-*b*:6,7-*b*′]dithiophene-4,5,9,10-tetracarboxylic acid diimide (8) (742 mg, 1 mmol) in dichloromethane (50 mL) at 0 °C. After stirring for 2 h, the mixture was diluted with methanol (200 mL). The resulting precipitate was collected by filtration and recrystallized from chloroform to give *N*,*N*′-bis(2-cyclohexylethyl)-2,7-dichloronaphtho[2,3-*b*:6,7-*b*′]dithiophene-4,5,9,10-tetracarboxylic acid diimide (**4**) as a red solid (253 mg, 38%): ^1^H-NMR (400 MHz, TCE-*d*_2_) δ 8.97 (s, 2H), 4.43 (t, *J* = 8.0 Hz, 4H), 1.98–1.75 (m, 14H), 1.64–1.56 (m, 2H), 1.46–1.20 (m, 10H); EI-MS (70 eV) = 667 (M^+^); Mp 337–338 °C; HRMS (EI) *m/z* calcd for C_34_H_32_Cl_2_N_2_O_4_S_2_ [M]^+^ 666.1181. Found: 666.1179; IR (KBr) ν = 1645, 1686 cm^−1^ (C=O). Due to the low solubility of 4, ^13^C-NMR signals were unclear even after 20,000 scans.

### 4.2. Single-Crystal X-ray Analysis

Single crystals of **3** and **4** suitable for X-ray structural analysis were obtained by recrystallization from chlorobenzene. The X-ray crystal structure analyses were carried out on a Rigaku R-AXIS RAPID (Cu Kα radiation, λ= 1.54187 Å, graphite monochromator, *T* = 100 K (**3**) or 90 K (**4**)). The structure was solved by direct methods. Non-hydrogen atoms were refined anisotropically using the SHELXL-2014 program [33], and hydrogen atoms were included in the calculations but not refined. The cif files of **3** and **4** are available in Supplementary Materials.

Crystallographic data for **3**: C_34_H_34_N_2_O_4_S_2_ (598.77), red pillar, 0.40 × 0.10 × 0.05 mm^3^, monoclinic, space group, *P*2_1_/*n* (#14), *a* = 16.370(4), *b* = 5.455(2), *c* = 17.688(4) Å, β = 116.037(9)°, *V* = 1419.2(6) Å^3^, *Z* = 2, *R*(*F*) = 0.1304 for 929 observed reflections (*I* > 2σ(*I*)) and 190 variable parameters, *wR*(*F*^2^) = 0.3434 for all data.

Crystallographic data for **4**: C_34_H_32_Cl_2_N_2_O_4_S_2_ (667.64), red needle, 0.30 × 0.10 × 0.01 mm^3^, monoclinic, space group, *P*2_1_/*n* (#14), *a* = 14.349(3), *b* = 4.927(1), *c* = 22.110(6) Å, β = 107.11(1)°, *V* = 1493.9(7) Å^3^, *Z* = 2, *R*(*F*) = 0.0807 for 1194 observed reflections (*I* > 2σ(*I*)) and 225 variable parameters, *wR*(*F*^2^) = 0.2731 for all data.

### 4.3. Device Fabrication and Evaluation

TFT devices based on **3** and **4** were fabricated in a top-contact-bottom-gate (TCBG) configuration on a heavily doped n^+^-Si (100) wafer with a 200 nm thermally grown SiO_2_ (*C*_i_ = 17.3 nF·cm^−2^). The substrate surfaces were treated with octadecyltrichlorosilane (ODTS) as reported previously [34]. Thin films as the active layer were vacuum-deposited on the Si/SiO_2_ substrates maintained at 150 or 200 *°*C at the rate of 1 Å·s^−1^ under the pressure of ~10^−3^ Pa. On top of the organic thin film, gold films (80 nm) as drain and source electrodes were deposited through a shadow mask. For a typical device, the drain-source channel length (*L*) and width (*W*) are 40 μm and 3.0 mm, respectively. Characteristics of the TFT devices were measured at room temperature under ambient conditions with a Keithley 4200 semiconducting parameter analyzer. Field-effect mobility (μ_FET_) was calculated in the saturation (*V*_d_ = *V*_g_ = +60) of *I*_d_ using the following equation,

*I*_d_ = *C*_i_ μ_FET_ (*W*/*2L*) (*V*_g_ − *V*_th_)^2^

where *C*_i_ is the capacitance of the SiO_2_ dielectric layer, and *V*_g_ and *V*_th_ are the gate and threshold voltages, respectively. Current on/off ratio (*I*_on_/*I*_off_) was determined from *I*_d_ at *V*_g_ = 0 V (*I*_off_) and *V*_g_ = +60 V. The μ_FET_ data of **3**- and **4**-based devices were typical values from nine different devices.

### 4.4. Theoretical Calculations

Calculations of intermolecular transfer integrals of dimers selected from the packing structures elucidated by single-crystal X-ray analysis were performed with the density functional theory (DFT) methods by using the ADF (Amsterdam Density Functional) package [29] with the PW91 functional and the basis set of Slater-type triple-ζ plus polarization (TZP).

## Figures and Tables

**Table 1:** Electronic structures of 2-cyclohexylethyl-NDTI derivatives (**3** and **4**).

Compound	*E*_red_^onset^/V ^1^	*E*_LUMO_/eV ^2^	λ_onset_/nm	*E*_g_/eV ^3^
**3**	−0.35	−4.0	574	2.16
**4**	−0.27	−4.1	556	2.23
**1**	−0.37	−4.0	569	2.17
**2**	−0.28	−4.1	552	2.24

^1^ V vs. Ag/AgCl; ^2^ Electrochemical *E*_LUMO_s were estimated from *E* (eV) = –4.4 − *E*_onset_; ^3^ Estimated from the absorption onset.

## Supplementary Materials

The following are available online at: http://www.mdpi.com/1420-3049/21/8/981/s1, CIF files for **3** and **4**.
